# Genome sequences of 31 mycobacteriophages isolated on *Mycobacterium smegmatis* mc^2^155 at room temperature

**DOI:** 10.1128/mra.01086-23

**Published:** 2023-12-15

**Authors:** Kirk R. Anders, Antonio Abeyta, Christy C. Andrade, Carla Y. Bonilla, Amanda B. Braley, Alexandra G. Bratt, Kaya A. Duncan, Stephen G. Hayes, Ciara J. Robinson, Helen Smith-Flores, Ann-Scott H. Ettinger, William F. Ettinger, Marta M. Fay, Joseph Haydock, Sean K. McKenzie, Rebecca A. Garlena, Daniel A. Russell, Marianne K. Poxleitner

**Affiliations:** 1 Department of Biology, Gonzaga University, Spokane, Washington, USA; 2 Department of Biological Sciences, University of Pittsburgh, Pittsburgh, Pennsylvania, USA; DOE Joint Genome Institute, Berkeley, California, USA

**Keywords:** bacteriophage

## Abstract

We report the genome sequences of 31 mycobacteriophages isolated on *Mycobacterium smegmatis* mc^2^155 at room temperature. The genomes add to the diversity of Clusters A, B, C, G, and K. Collectively, the genomes include 70 novel protein-coding genes that have no close relatives among the actinobacteriophages.

## ANNOUNCEMENT

The bacteriophage population is enormous and genetically diverse (1). Sequencing and comparison of bacteriophage genomes has provided many insights in viral biology, diversity, and evolution (2, 3). To contribute to understanding the viruses of Actinobacteria, we isolated 31 phages on *Mycobacterium smegmatis* mc^2^155 as part of the Science Education Alliance-Phage Hunters Advancing Genomics and Evolutionary Science (SEA-PHAGES) program at Gonzaga University (4, 5).

Twenty-nine soil samples were collected in 2013–2017 from distinct locations in Spokane, WA, 1 from Olympia, WA (containing phage Jeeves) and 1 from Phoenix, AZ (containing Kristoff). The samples were incubated with *M. smegmatis* in Middlebrook 7H9 for 2–7 days, filtered at 0.22 µm, and plated onto lawns of *M. smegmatis*. Plaques were picked and re-plated at least three times before lysates were collected (6). All cultures were grown at room temperature (22°C). DNA was purified from lysates with the Promega Wizard DNA Clean-Up System. Sequencing libraries were prepared with NEB Ultra II FS kits (v.3 reagents), pooled, then sequenced by Illumina MiSeq, yielding single-end, 150-base reads (Table 1). The reads were assembled with Newbler (v.2.9) (7), producing average coverage depths from 89× (Zimmer) to 3,109× (Miko). Consed (v.28–29) was used to check the assemblies for completeness and accuracy and to determine the structures of genome termini (8, 9). Genome lengths were 44,842–164,254 bp; GC contents were 61.2%–69.3%; and three genomes were circularly permuted instead of sporting distinct ends (Table 1). Genes were predicted using Glimmer (v.3.02) (10), Genemark (v.2.5p) (11), Aragorn (v.1.2) (12), and tRNAscan-SE (v.1.3–2.0) (13), comparatively analyzed with Starterator (v.1.0–1.2) (https://github.com/cdshaffer/starterator), and Phamerator (14), then manually refined with DNA Master (v.5.22–5.23) (http://cobamide2.bio.pitt.edu). Gene functions were predicted using HHPred to search PDB (15), Pfam (16), SCOP (17), and CCD (18) databases within the HH-Suite (v.3.0–3.2) (19, 20), TmHMM (v.2.0) (21), DeepTMHMM (v.1.0) (https://dtu.biolib.com/DeepTMHMM), NCBI BLAST (v.2.2–2.10) (22), and the Actinobacteriophage Database (23). Default parameters were used for all software. The genomes carried 66–244 protein-coding genes, and most contained at least one tRNA (Table 1). Phabba, the largest genome, contained 37 tRNAs and 1 tmRNA.

**TABLE 1:** Genome characteristics and accession numbers of 31 mycobacteriophages

Phage name	Cluster	No. of reads	Genome length (bp)	GC content^*a*^ (%)	Genome end^*b*^	Overhang sequence	No. of protein-coding genes	No. of tRNAs	No. of orphams^*c*^	SRA^*d*^ accession no.	GenBank accession no.	Location
Chargerpower	A	421,744	52,886	61.5	3′ 10-base ext.	CGGTCGGTTA	100	1	5	SRX21748139	OR159676	47.666034 N, 117.404057 W
Eyeball	A1	1,482,846	52,231	63.9	3′ 10-base ext.	CGGATGGTAA	94	–	–	SRX21748145	OM203162	47.667374 N, 117.402246 W
Kristoff	A10	746,221	51,236	65.0	3′ 10-base ext.	CGGCCGGTAA	83	1	–	SRX21748115	MK494124	33.650697 N, 111.975133 W
DarthPhader	A12	1,089,744	53,432	63.2	3′ 10-base ext.	CGGGCCGTAA	91	1	2	SRX21748143	KX657793	47.666276 N, 117.402764 W
Zimmer	A12	17,794	53,048	63.1	3' 10-base ext.	CGGGACGTAA	90	1	–	SRX21748140	OM203158	47.667314 N, 117.402271 W
Jeeves	A14	531,177	52,466	62.1	3' 10-base ext.	CGGTCGGTTA	101	1	1	SRX21748112	MN310541	47.076319 N, 122.829517 W
SororFago	A14	39,847	53,516	62.1	3' 10-base ext.	CGGTCGGTTA	97	1	–	SRX21748135	OM203161	47.667137 N, 117.403002 W
Iwokeuplikedis	A2	694,892	53,261	62.7	3' 10-base ext.	CGGTCGGTTA	98	1	2	SRX21748111	MT310858	47.666944 N, 117.402778 W
Miko	A2	1,152,302	51,881	63.5	3' 10-base ext.	CGGTCGGTTA	90	1	-	SRX21748123	MN369748	47.666456 N, 117.404914 W
HarryHoudini	A2	74,786	52,722	63.4	3' 10-base ext.	CGGTCGGTTA	95	1	1	SRX21748108	OM203159	47.667778 N, 117.398060 W
Phillis	A8	33,855	51,427	61.2	3' 10-base ext.	CGGGATGTAA	97	1	2	SRX21748126	MZ322005	47.664860 N, 117.403198 W
Charm	A9	745,074	52,596	61.9	3' 10-base ext.	CGGTCGGTTA	92	2	-	SRX21748141	MK494108	47.668611 N, 117.401944 W
LoneWolf	A9	476,243	53,186	63.0	3' 10-base ext.	CGGTCGGTTA	98	1	-	SRX21748119	MN369741	47.667359 N, 117.402248 W
Rahalelujah	A9	951,660	52,816	62.4	3′ 10-base ext.	CGGTCGGTTA	97	1	1	SRX21748129	MN369764	47.667059 N, 117.402813 W
Frankicide	B1	569,210	68,266	66.5	Circularly perm.		99	-	-	SRX21748107	OR475293	47.668040 N, 117.403456 W
LilMcDreamy	B12	1,033,803	71,552	69.3	Circularly perm.		99	-	11	SRX21748118	MN284893	47.667370 N, 117.402245 W
Phabba^*e*^	C2	523,822	164,254	65.2	Circularly perm.		244	37 + 1 tmRNA	33	SRX21748125	MF668280	47.667702 N, 117.402889 W
Pinnie	G3	859,247	44,842	68.8	3′ 10-base ext.	CTCGTGGCAT	66	-	1	SRX21748127	MK494105	47.667189 N, 117.402375 W
Chris	K1	530,143	62,067	67.2	3′ 11-base ext.	CTCGTAGGCAT	100	1	4	SRX21748142	MT310860	47.667540 N, 117.400587 W
KiSi	K1	736,467	62,558	68.7	3′ 11-base ext.	CTCGTAGGCAT	103	1	-	SRX21748114	MK376955	47.667074 N, 117.402995 W
LeMond	K1	391,192	62,515	68.8	3′ 11-base ext.	CTCGTAGGCAT	102	1	-	SRX21748116	MH910038	47.665923 N, 117.403924 W
MarkPhew	K1	512,205	62,153	67.9	3′ 11-base ext.	CTCGTAGGCAT	102	1	1	SRX21748121	MT310859	47.666537 N, 117.403032 W
Nibb	K1	661,146	62,293	67.6	3′ 11-base ext.	CTCGTAGGCAT	102	1	1	SRX21748124	MK460246	47.667233 N, 117.402333 W
Scarlett	K1	1,070,962	62,306	68.7	3′ 11-base ext.	CTCGTAGGCAT	102	1	-	SRX21748133	MH910042	47.667062 N, 117.403006 W
Heftyboy	K5	439,326	61,968	65.0	3′ 11-base ext.	CTCAGTGGCAT	95	1	-	SRX21748110	OR475256	47.666811 N, 117.404644 W
SoSeph	K5	95,424	61,968	65.0	3′ 11-base ext.	CTCAGTGGCAT	95	1	-	SRX21748136	MZ322016	47.668056 N, 117.403611 W
Shadow1	K6	425,281	60,455	66.7	3′ 10-base ext.	CTCGGGGCAT	100	-	-	SRX21748134	OR475277	47.666903 N, 117.402809 W
Syra333	K6	482,790	60,295	66.7	3′ 10-base ext.	CTCGGGGCAT	99	-	-	SRX21748137	OR434023	47.666944 N, 117.402515 W
Tierra	K6	357,243	61,418	66.3	3′ 10-base ext.	CTCGGGGCAT	101	1	-	SRX21748138	OR475288	47.666389 N, 117.402778 W
Bryler	K6	660,575	57,666	66.1	3′ 10-base ext.	CTCGGGGCAT	92	2	1	SRX21748128	MN369762	47.667074 N, 117.402995 W
Marshawn	K6	614,204	61,464	68.1	3′ 10-base ext.	CTCGTGGCAT	95	1	4	SRX21748122	MN284895	47.666330 N, 117.404649 W

^*a*^ The host genome, *Mycobacterium smegmatis* mc^2^155 (GenBank accession number NC_008596 .1), has a GC content of 67.4%.

^*b*^ ext, single-stranded overhanging extension; perm., permuted.

^*c*^ orpham, protein-coding gene with no close relatives in the Actinobacteriophage Database, as determined by Phamerator (14), using the set of all proteins in the Actino_Draft database, v.522, Aug 2023.

^*d*^ SRA, Sequence Read Archive.

^*e*^ Myovirus, based on transmission electron microscopy (TEM) and Cluster C type genome. All other phages are siphoviruses, based on TEM and/or cluster membership.

**Fig 1:**
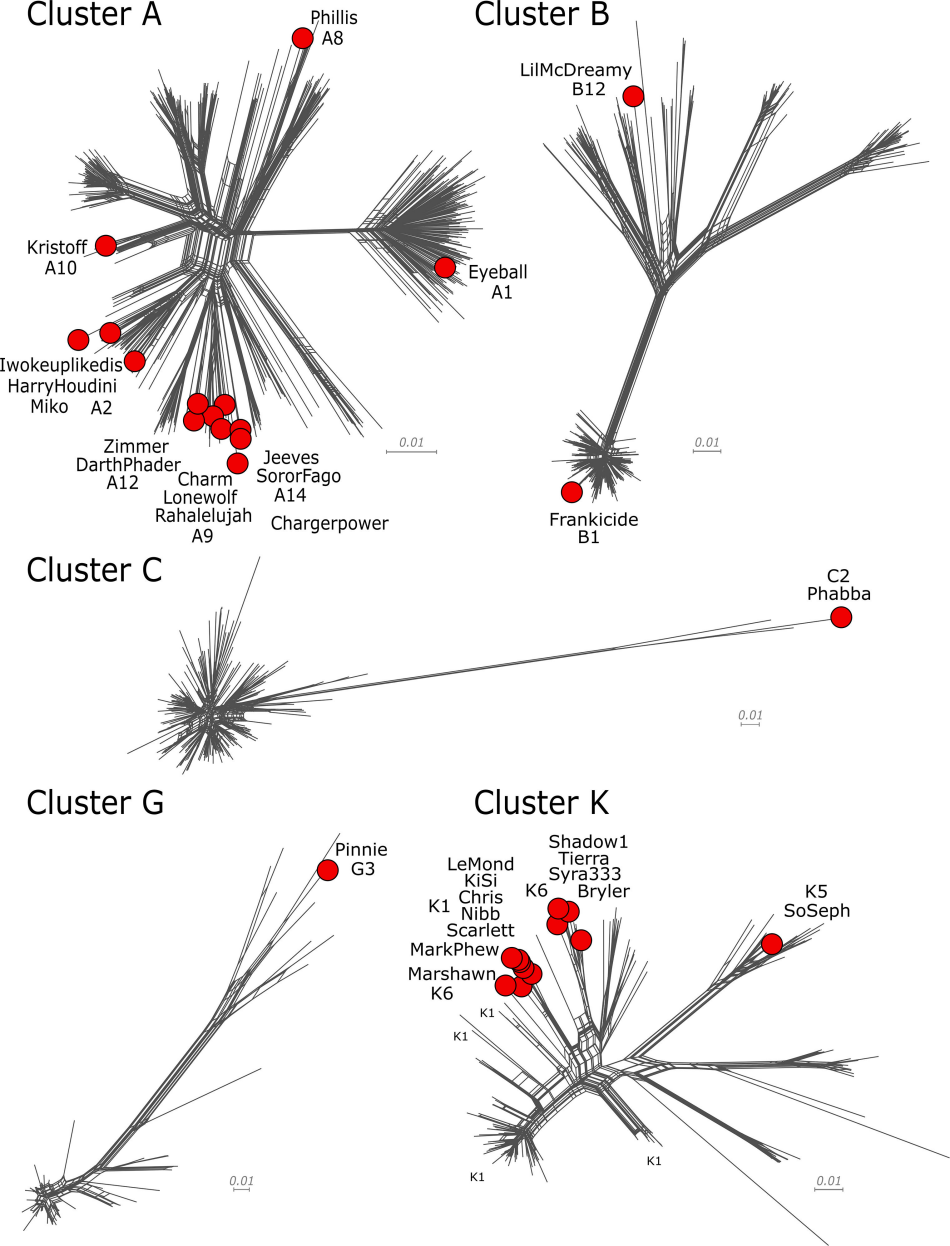
Relationships among the 31 phages and all the phages of Clusters A, B, C, G, and K. Network trees of shared gene content are displayed, showing relationships among the phages within each cluster. The branch for each phage described in this report is marked with a red circle at its end, and its name and subcluster membership are indicated. To generate the trees, all protein-coding genes in the Actinobacteriophage Database (Actino_Draft, v.521) were grouped into families (phams) with Phamerator (14) based on amino acid sequence similarities. Each genome in a cluster was then scored according to the presence or absence of each pham, and the distance between each phage was computed by SplitsTree (v.4.19) (25), which generated an unrooted network representation of the relationships. The scale bars indicate 0.01 substitutions/site.

The genomes were grouped with five actinobacteriophage clusters (Clusters A, B, C, G, and K) based on gene content similarity (14, 24), and all but Chargerpower were assigned to 1 of 14 subclusters based on average nucleotide identity determined by DNA Master (2) (Table 1; Fig. 1). The genomes were not evenly distributed across clusters (Fig. 1). Fourteen genomes joined Cluster A, 13 joined Cluster K, but only 1 genome each was placed in Cluster C (Phabba, Subcluster C2) and Cluster G (Pinnie, G3). LeMond, KiSi, Chris, Nibb, Scarlett, and MarkPhew, along with Oscar (GenBank accession MH910039), shared 84%–98% gene content and defined a distinct branch on a Cluster K tree (Fig. 1). LilMcDreamy (B12) was the most distantly related genome in the group, sharing only 66% of its genes with its nearest neighbor, Reprobate (B5, GenBank accession KF024727). Half of the genomes carried at least one *orpham*, a gene with no close relatives among actinobacteriophages (14), with Phabba carrying the most orphams at 33 (Table 1).

## Data Availability

Thirty-one whole-genome shotgun projects have been deposited in DDBJ/ENA/GenBank under the GenBank and Sequence Read Archive accession numbers listed in Table 1. The GenBank genome versions described in this paper are the first versions, each denoted as the GenBank number in Table 1 followed by “.1.”
